# Research Progress on the Mechanism and Application of the Type I CRISPR-Cas System

**DOI:** 10.3390/ijms252312544

**Published:** 2024-11-22

**Authors:** Peihong Yang, Shuai Zhang, Debao Hu, Xin Li, Yiwen Guo, Hong Guo, Linlin Zhang, Xiangbin Ding

**Affiliations:** Key Laboratory of Animal Breeding and Healthy Livestock Farming, College of Animal Science and Veterinary Medicine, Tianjin Agricultural University, Tianjin 300392, China; 2203010121@stu.tjau.edu.cn (P.Y.); 2203010124@stu.tjau.edu.cn (S.Z.); hudebao@tjau.edu.cn (D.H.); lixin82@tjau.edu.cn (X.L.); yiwenguo@tjau.edu.cn (Y.G.); guohong@tjau.edu.cn (H.G.)

**Keywords:** type I CRISPR-Cas system, mechanism of action, gene editing

## Abstract

The CRISPR-Cas system functions as an adaptive immune mechanism in archaea and bacteria, providing defense against the invasion of foreign nucleic acids. Most CRISPR-Cas systems are classified into class 1 or class 2, with further subdivision into several subtypes. The primary distinction between class 1 and class 2 systems lies in the assembly of their effector modules. In class 1 systems, the effector complex consists of multiple proteins with distinct functions, whereas in class 2 systems, the effector is associated with a single protein. Class 1 systems account for approximately 90% of the CRISPR-Cas repertoire and are categorized into three types (type I, type IV, and type III) and 12 subtypes. To date, various CRISPR-Cas systems have been widely employed in the field of genetic engineering as essential tools and techniques for genome editing. Type I CRISPR-Cas systems remain a valuable resource for developing sophisticated application tools. This review provides a comprehensive review of the characteristics, mechanisms of action, and applications of class 1 type I CRISPR-Cas systems, as well as transposon-associated systems, offering effective approaches and insights for future research on the mechanisms of action, as well as the subsequent development and application of type I CRISPR-Cas systems.

## 1. Introduction

CRISPR-Cas (Clustered Regularly Interspaced Short Palindromic Repeats and CRISPR-associated proteins) is a widely occurring adaptive immune system found in bacteria and archaea. It is mediated by RNA molecules that protect against external nucleic acid invasions. The advent of this system has significantly accelerated the development of gene-editing technologies, as researchers have elucidated its underlying principles and structures, subsequently engineering it into novel, precise gene-editing tools. Type I CRISPR systems have become widely studied and applied in genome editing in recent years. Interestingly, researchers have found that many of these systems can produce large deletions and even bidirectional deletions. Therefore, optimizing the use of type I CRISPR systems for more efficient genome editing has become a current research focus. This review first introduces the classification and mechanisms of the type I CRISPR-Cas system. It then provides a concise summary of the history, characteristics, research findings, and validated gene-editing efficiencies of various isoforms, along with their applications in the field of gene editing. Finally, we explore the potential of type I CRISPR-Cas systems for enabling gene editing in both prokaryotic and eukaryotic organisms in the future.

## 2. Overview of the CRISPR-Cas System

Since the discovery of the CRISPR-Cas system, researchers have been studying it, exploring its mechanisms, and adapting it into a powerful gene-editing tool that can be used. Currently, the RNA-guided nuclease in the CRISPR-Cas system is considered the most reliable tool for genome editing and engineering [1]. The history of the discovery of CRISPR is very interesting. In 1987, Nakata et al. studied the *IAP* gene of *E. coli* and found that there was a set of repetitive sequences downstream of it, which contained a non-repetitive sequence in the middle of the repetitive sequences [2]. However, the function of these repetitive sequences was initially unclear. Subsequent studies found that these sequences are common in the genomes of bacteria and archaea and were officially named CRISPR in 2002, and the characterized genes found near these sequences were named CRISPR-associated genes (Cas) [3].

In recent years, CRISPR-Cas9 has been developed in various forms as a genome-editing tool and technology in the field of genetic engineering, and most CRISPR-Cas systems are categorized as either class 1 or class 2 and further divided into several subtypes within each class [4]. Class 1 systems make up about 90% of the CRISPR-Cas body and are found in various bacteria and archaea. However, class 2 systems are much less abundant in nature, accounting for only about 10% of all CRISPR systems found in sequenced microbial genomes. The main difference between class 1 and class 2 systems is in the way the effector modules are assembled. In class 1 systems, effectors are composed of multiple proteins with different functions, whereas in class 2 systems, effectors are associated with only one protein [5,6]. Based on the different effector protein families, class 1 systems are classified into three types (type I, type III, and type IV) and 12 subtypes [7]. Type II systems have a relatively simple composition; therefore, researchers have thoroughly examined their structures and underlying concepts. While the composition and mode of action of type III and type IV systems have received less research attention among type I systems, type I systems are currently a popular area of study and are generally more well understood.

**Figure 1:**
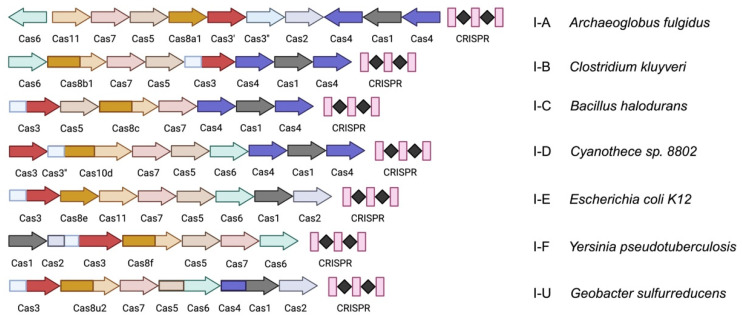
Information on representative strains and gene clusters for each isoform of the type I CRISPR-Cas system. The figure illustrates the representative strains and gene clusters associated with different isoforms of the Type I CRISPR-Cas system. Pink rectangles denote repeats, while black diamonds represent spacers between repeats. Different colored arrows represent distinct Cas proteins, highlighting the diversity of gene clusters within the Type I system.

DNA helicase Cas3, which is involved in cutting and degrading DNA, exhibits deconjugating enzyme activity and single-stranded DNA nuclease activity and is one of the many proteins constituting type I systems [8]. Other effector proteins combine to form a multisubunit ribonucleoprotein (RNP) complex known as Cascade (CRISPR-associated antiviral defense complex). Besides effector proteins, most type I systems encode adaptor module proteins Cas1 and Cas2, along with various helper proteins like reverse transcriptase, CARF (CRISPR-associated Rossmann fold) domain-containing proteins, and others [7]. The integration of the new spacer sequences is facilitated by the Cas1, Cas2, and Cas4 proteins [9,10,11,12,13,14]. The composition of Cas proteins further categorizes type I systems into seven isoforms, from I-A to I-F and I-U (Figure 1) (Table 1) [15,16]. The primary proteins in this system include Cas1, Cas2, Cas3, Cas4, Cas5, Cas6, Cas7, Cas8, and Cas10d, though the names of the various isoforms may differ.

## 3. Mechanisms of Type I CRISPR-Cas Systems

**Figure 2:**
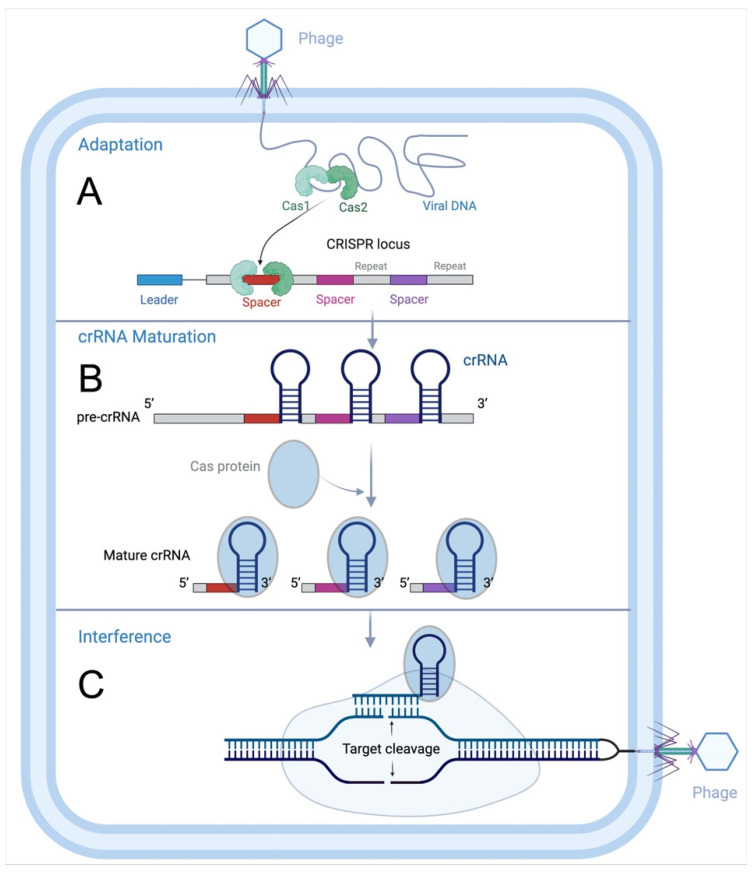
The three stages of CRISPR immunity are as follows: (**A**) Adaptation: The Cas1-Cas2 complex recognizes and selects a segment of foreign DNA, which is subsequently integrated into the host’s CRISPR array. (**B**) crRNA Maturation: The CRISPR array is transcribed into a long pre-crRNA, which is then further processed by Cas proteins to produce a mature crRNA. (**C**) Interference: The mature crRNA guides the Cas nuclease to the homologous foreign DNA. Upon binding of the crRNA to the target sequence, Cas proteins cleave the foreign nucleic acid.

**Figure 3:**
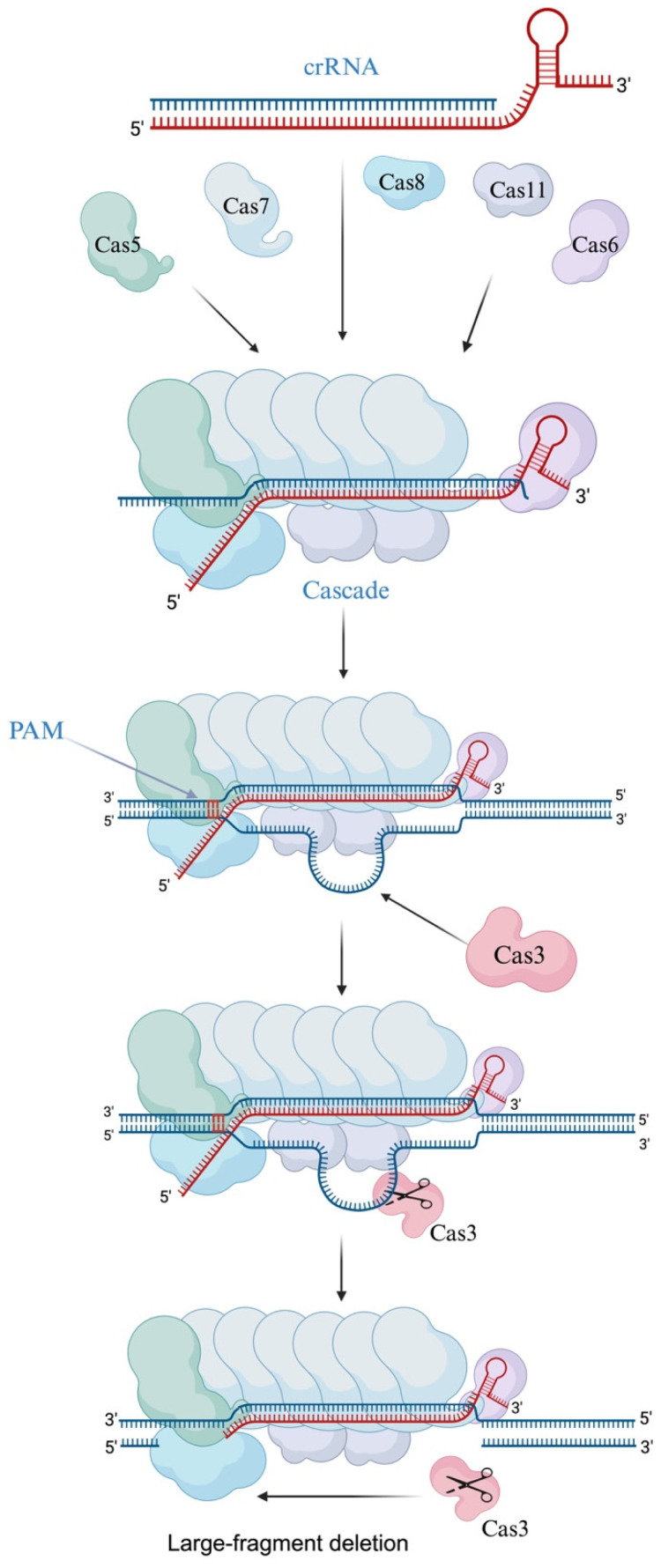
Mechanism of cleavage in the type I CRISPR-Cas system (example: type I-E): The cleavage process begins with the assembly of individual Cas proteins into the Cascade. In this complex, Cas6 binds to the crRNA and anchors the 3’ hairpin, while Cas5 binds to the 5’ hairpin. Cas7 forms the backbone along the crRNA, and Cas11 constitutes the core of the Cascade. Cas8, which recognizes the PAM, contains a specific domain that can directly interact with Cas3. This interaction facilitates the recruitment of Cas3 to the target DNA site and stabilizes its spatial positioning to enable efficient cleavage. Cas3 is specifically recruited to the Cascade and activated only when the crRNA fully complements the target strand, forming a complete R-loop. Upon activation, Cas3 cleaves the non-target DNA strand at the displaced DNA, resulting in a large deletion.

Three phases are necessary for CRISPR immunization in type I systems, as they are in other systems: an adaptation phase, a CRISPR RNA (crRNA) maturation phase, and an interference phase (Figure 2). Additionally, each of them needs a protospacer adjacent motif (PAM), which differs depending on the isoform and is found at 5′ or 3′ of the (proto)spacer [7]. The type I system has a different interference mechanism than Cas9. Through a series of steps, it often leads to large-fragment deletions instead of precise DSBs. First, Cascade employs the 5’ in PAM in the genome to identify the target site using CRISPR RNA (crRNA) [17,18,19]. If the target DNA is correctly matched to the crRNA, a stable R-loop is formed, and large-scale conformational changes are triggered [19,20,21]. The helicase–nucleic enzyme Cas3 is then specifically recruited to the R-loop to form a Cascade [22], which cleaves the non-target strand (NTS) DNA and sustains degradation of its upstream region near the PAM site [20]. Cas3 then continues to degrade target strand (TS) DNA; however, the subsequent mechanisms of this process may vary depending on the specific Cas3 subtype [15]. The backbone part of the effector complex Cascade generally consists of Cas7, Cas5, and Cas6 proteins or their homologs [21]. Cas7 proteins have a copy number of 6–7, and a number of them make up the Cascade’s core, which binds and sustains crRNA and modifies how it binds to DNA; Cas5 proteins are linked to substrate nucleic acid binding and have a low relative molecular mass. One of the most common families of Cas proteins in the CRISPR-Cas system is Cas5 proteins, which are found in large quantities in type I-A, type I-B, type C, and type E systems. In the I-C system, Cas5 proteins immobilize the 5′ end of crRNA and aid in the maturation of pre-crRNA [23]. The Cas6 protein exhibits RNA endonuclease activity, binding to the hairpin structure formed by a portion of the repeat sequence. It cleaves the long pre-crRNA to generate mature crRNA and remains stably associated with the hairpin structure post-cleavage. This association protects the crRNA from further degradation. Subsequently, Cas6 forms a complex with other proteins, securing the 3′ end of the crRNA [24]. However, the Cas5 protein in the I-C isoform is responsible for developing pre-crRNA [25]. Furthermore, during substrate DNA binding, the major subunits of the complex, Cas10d, Cas8, and Cse1, recognize PAM sequences, and their structural domains connect directly with the Cas3 protein to stabilize Cas3’s spatial position. With a comparatively low molecular mass, Cas11 (Cse2) is engaged in binding and immobilizing dsDNA substrate non-target strands (Table 2) (Figure 3). They are extensively employed in the manipulation of microbial genomes, encompassing Cascade-based transcriptional regulation, Cascade-Cas3-based programmable antimicrobials, natural variant selection, gene editing assisted by homologous recombination (HR), genome minimization, and targeted DNA integration [26,27,28,29,30]. Large-fragment deletions are the primary characteristic of type I CRISPR-Cas systems. Following the successful integration of crRNA by the Cascade, Cas3 can be recruited to carry out continuous DNA degradation and complete large-fragment cleavage of genomic DNA through multi-protein synergy. Type I-C and type I-E systems are now the most researched [17,20,31,32,33]. Only recently has the type I CRISPR-Cas system been successfully used in eukaryotes.

Cas3 is a multifunctional protein with deconjugating enzyme activity and single-stranded DNA nuclease activity, performing the primary cleavage function. Cas3 is specifically recruited to the Cascade complex only when the crRNA is fully complementary to the target DNA, resulting in the formation of a complete R-loop. Upon recruitment, Cas3 is activated and cleaves the displaced DNA strand generated by the non-target DNA strand.

## 4. Gene-Editing Applications of the Type I CRISPR-Cas System

The CRISPR-Cas9 system was one of the first systems discovered by researchers, and its molecular mechanism has been thoroughly investigated. It has now become the gene-editing tool of choice for biomedical and genetic research [37]. But despite its simplicity and ease of use, there are certain related issues. For example, following gene knockout using the CRISPR-Cas9 system, residual pseudo-mRNA may still encode proteins. Pseudo-mRNA refers to mRNA transcribed from repaired DNA sequences after gene knockout, which can still retain coding potential. These pseudo-mRNAs can be translated into functional proteins, potentially affecting the outcome of gene editing [38]. Moreover, the commonly used Cas9 and Cas12 systems have limited capability for generating large deletions. While Cas12 can often generate substantial deletions (typically greater than 3 bp) that may cause frameshift mutations, deletions in multiples of three could leave the resulting protein functionally intact. Consequently, in applications requiring extensive sequence deletions, Cas9 and Cas12 may fall short of achieving the desired editing effect. In contrast, large-fragment deletions caused by the type I CRISPR system are difficult to repair, which differentiates it from Cas12. The type I system may address this limitation and could be developed into a more effective gene-editing tool. Increasingly, researchers have shifted their focus toward type I systems to study gene-editing efficiency at the cellular level in both prokaryotic and eukaryotic cells, advancing gene-editing research (Table 3).

### 4.1. I-A CRISPR System

The type I-A CRISPR system was the first of the type I systems to be discovered. Unlike the others, it was isolated from the thermophilic archaeon *Thermoproteus tenax*, which thrives at temperatures up to 86 °C [49]. *T. tenax* contains nine Cas proteins that recombine to form two complexes: the CRISPR-associated complex for the integration of spacers (Cascis), thought to mediate spacer integration, and a six-protein extended Cascade-like complex [50]. The presence of *Csa1* close to the *Cas4*, *Cas1*, and *Cas2* genes is the primary characteristic of the CRISPR-Cas system of the I-A subtype. Moreover, *Cas3* is located near to the genes *Csa5*, *Cas8a*, *Cas5*, *Cas7*, and *Cas6* and is split into two distinct genes, *Cas3* and *Cas3*′ [51]. Remarkably, the Cas3 protein in the type I-A CRISPR system is initially coupled to Cascade and does not need to be re-engaged by means of Cascade’s recognition of the target. Cas4 nuclease was revealed to be crucial for pre-spacer processing and PAM selection in the type I-A CRISPR system from *Sulfolobus solfataricus* [52].

F. He et al. investigated how the CRISPR-Cas system is triggered by viral infection or how its expression is managed when there is no viral infection. They found that transcriptional repression is maintained by both the Csa3b and Cascade complexes binding to the promoter region of the CRISPR-Cas type I-A interferon cassette. Redistribution of the Cascade complex into the crRNA-matched protospacer after viral infection alleviated transcriptional inhibition, which in turn decreased the virus’s ability to attach to its promoter and effectively impede its spread [53]. S. Majumdar and M. P. Terns resolved the assembly architecture and mechanism of action of the type I-A crRNPs and found that the type I-A DNA cleavage of crRNPs requires crRNA-directed and in situ adjacent motif-dependent binding of target DNA to unwind double-stranded DNA and expose the single strand for ATP-dependent 3′–5′ cleavage catalyzed by Cas3′ helicase and Cas3″ nuclease crRNP components [54]. 

C. Hu et al. engineered a modified Pfu Cascade-Cas3 system to develop a temperature-activated, low-background, and highly sensitive nucleic acid detection tool. When the correct target is present, the activation of Cas3 triggers an accessory activity that cleaves the FQ-ssDNA probe (fluorescence-quenched single-stranded DNA probe), resulting in fluorescence. The intensity of the fluorescence can then be used to determine the presence or absence of the target DNA. In this subtype, the Cascade-Cas3 complex exhibits no nuclease activity when not bound to the target DNA. Based on this concept, the researchers created a suite of rapid and efficient nucleic acid molecular diagnostic tools named “HASTE” (Heat-Activated Streamlined Nucleic Acid Detection Platform). Reports indicate that this technology can detect DNA at the single-molecule level, delivering impressive results in less than 15 min. It offers reliability and accuracy comparable to traditional PCR but at a significantly lower cost and with reduced time requirements. Additionally, in this system, Cas3’s deconjugating enzyme activity is strictly temperature-dependent, with enhanced activity at higher temperatures. Utilizing a temperature stimulus of 42 °C, the team developed a set of temperature-controlled gene knockout tools with an efficiency exceeding 90% [39]. Therefore, in the future, this property could be leveraged to regulate the gene-editing efficiency of this tool through localized temperature stimulation, thereby reducing the off-target effects associated with gene-editing tools. However, there are limitations to this tool, as its deletion editing is primarily dependent on temperature, requiring conditions of 42 °C to achieve optimal results. Given the unique characteristics of the I-A system, could it be applied to gene editing in eukaryotic cells? Subsequently, researchers delivered the Cascade-Cas3 complex as an RNP complex into HAP1 cells via electroporation. Exploiting its temperature-dependent editing properties, they observed editing efficiencies of up to 90% under 42 °C. Interestingly, they also discovered its bidirectional deletion capability [39].

Additionally, T. Hu and colleagues discovered a novel type I-A Cas3 variant from *Thermococcus siculi*, which remains in a self-inhibitory state when not triggered, effectively reducing non-specific background noise. They subsequently applied this variant to nucleic acid detection and developed a hyperactivity verification device (HAVE). This system enables the rapid and accurate diagnosis of human papillomavirus (HPV) in clinical samples. In the detection process, the sample first undergoes recombinase polymerase amplification (RPA) at 37 °C for 10 min to amplify the target nucleic acid, making detection easier. The RPA product is then mixed with TsiCascade-Cas3 and an F-Q (fluorophore-quencher) ssDNA reporter molecule, and the reaction is performed at 85 °C for 15 min. If the target nucleic acid is present, the nuclease activity of Cas3 will be activated, leading to the cleavage of the F-Q reporter molecule and the release of a fluorescent signal. The results can be directly read using a portable fluorescence device [55].

### 4.2. I-B CRISPR System

In 2013, L. K. Maier, S. J. Lange et al. investigated the CRISPR-Cas type I-B system in the halophilic archaebacterium *Haloferax volcanii* [56]. Eight Cas proteins (Cas1–Cas8b) make up the Haloferax system, which shows that CRISPR-Cas type I-B also needs seed sequences to engage with invaders [57,58]. The recombinant complexes and *C. thermocellum* cell lysates have a short C-terminal Cas8b fragment. The CRISPR-Cas type I-B complexes of *C. thermocellum* and *C. maripaludis Methanococcus* comprise Cas7, Cas5, Cas6b, and the big subunit Cas8b, which consists of two tightly bound protein pieces [59,60]. The fact that this approach is operational in six distinct PAM sequences—TTC, ACT, TAA, TAT, TAG, and CAC—makes target selection easier and more flexible when using it [61]. According to research by A. Maikova et al., recently acquired spacers prefer the proto-spacer linked to interference-competent PAMs. This finding also demonstrates the functional relationship between interference and adaptive mechanisms [62].

For gene editing in prokaryotes, M. E. Pyne et al. discovered that the native type I-B CRISPR-Cas system produced up to 100% gene-editing efficiency in *Clostridium pasteurianum* [63]. However, the endogenous type I-B CRISPR-Cas system in the halophilic archaeon *Haloferax volcanii* demonstrates a high degree of self-targeting tolerance [64], which renders the system challenging to harness. Researchers speculate that this high tolerance may be attributed to the high copy number of chromosomes within the *Haloferax volcanii* cells, which facilitates rapid and accurate repair through homologous recombination (HR) [65,66]. F. Cheng et al. have successfully applied the native type I-B CRISPR-Cas system for precise genomic editing in the polyploid archaeon *Haloarcula hispanica*, optimizing the gene-editing tool to carry a single plasmid that can simultaneously accommodate self-targeting mini-CRISPR and a donor of 600–800 bp [67]. This study successfully demonstrated the potential for one-step simultaneous editing of two loci, providing valuable insights and references for genome editing in other archaea, particularly in polyploid archaea. J. E. Walker et al. have developed a novel tool that enhances the efficiency of CRISPR-Cas genome editing in the thermophilic cell *C. thermocellum*, increasing the editing efficiency of the type I-B system from 14% to 70% [68]. K. Dai et al. have employed the endogenous type I-B CRISPR-Cas system to successfully delete the *ldh* and *argR* genes in the *Thermoanaerobacterium aotearoense* strain SCUT27, achieving an editing efficiency ranging from 58.3% to 100%, thereby enhancing ethanol production from lignocellulosic hydrolysates [69]. Yang et al. have identified a thermostable type I-B CRISPR-Cas from *P. thermoglucosidasius* that can cleave target DNA using full-length complementary crRNA and have demonstrated the capability of this thermostable I-B system for efficient genome editing and gene repression in neutralophilic bacterial species [40]. In summary, in future medical research, this system could potentially be used to study the pathogenic mechanisms of microorganisms and develop new prevention and treatment strategies, such as reducing pathogen virulence or enhancing vaccine efficacy through gene editing.

In the domain of gene editing for eukaryotic organisms, M. Lu et al. have utilized cryo-electron microscopy to reconstruct the Syn I-B-type Cascade, identifying the optimal PAM sequence as 5’-AYG-3’. This fills an important gap in the field. Notably, both Cas5b and Cas8b are implicated in PAM recognition. By introducing the Syn I-B-type CRISPR system into human CD3+ T cells, they achieved a remarkable gene-editing efficiency of up to 41.2% and induced a unidirectional genomic deletion spanning 4.5 kb [70].

### 4.3. I-C CRISPR System

In 2012, researchers elucidated the structural composition and molecular mechanisms of the I-C CRISPR-Cas system from *Bacillus halodurans*, which encodes the proteins Cas5, Cas7, Cas8, and Cas3 [71,72]. The specific protein Cas5d from *B. halodurans* functions as a pre-crRNA endonuclease, recognizing stem–loop repeat sequences with a specific 3’ adjacent region, cleaving at the 3’ end of the hairpin to produce crRNA, and assembling with crRNA into an interference complex similar to *E. coli*’s Cascade [72]. In addition to being an RNA endonuclease, Cas5d also possesses metal-dependent DNase activity, with the DNase and RNase active sites seemingly overlapping [73]. The I-C CRISPR-Cas system, known for its compact structure and remarkable gene-editing efficiency, has been extensively studied. Research shows that the I-C-type Cas3 protein from *Neisseria lactamica* (Nla) is composed of three primary domains: an HD nuclease domain, a RecA-like helicase domain, and a C-terminal DNA-binding domain (CTD). Cas3 utilizes the energy generated by ATP hydrolysis within the RecA-like helicase domain to unwind double-stranded DNA (dsDNA) into single-stranded DNA (ssDNA), which is subsequently cleaved by the HD nuclease domain [74]. Understanding the activation mechanism of the Cas3 protein is crucial for designing more effective gene-editing tools and strategies.

To develop the I-C CRISPR-Cas system as a convenient gene-editing tool, researchers identified the I-C-type CRISPR-Cas system in the genome of *Xanthomonas oryzae pv. oryzae* (Xoo), determining the PAM sequence for this subtype to be 5’-TTC-3’, and demonstrated that a gRNA of at least 27 bp in length, directly matching the downstream sequence of the TTC PAM, is a necessary condition for effective CRISPR function [75]. D. Jiang et al. utilized the endogenous I-C-type CRISPR-Cas system of Xoo to achieve diverse genomic editing outcomes, efficiently inducing large genomic deletions of up to 212 kb [76]. W. E. Geslewitz et al. introduced the DNA-binding module of the I-C-type CRISPR-Cas from Nla into the transcriptionally silent intergenic region of the Neisseria gonorrhoeae (Gc) chromosome, developing a novel, inducible, minimal CRISPR interference (CRISPRi) system for gene-specific transcription repression in Gc, and demonstrated that the I-C CRISPRi system can conditionally reduce the expression of two essential genes [77]. B. Csorgo et al. repurposed and optimized the I-C-type CRISPR system of Pseudomonas aeruginosa, using a single crRNA to produce genome-wide large deletions with nearly 100% efficiency (up to 424 kb), and found that it can generate bidirectional deletions [27]. Renke Tan et al. employed the I-C-type CRISPR-Cas system of Nla to generate targeted large deletions in the human genome, observing an editing efficiency of approximately 95%. Moreover, the assembly of NlaCascade in bacteria requires the translation of a hidden component Cas11 from within the Cas8 gene, and the expression of separately encoded NlaCas11 in human cells is key to achieving plasmid and mRNA-based editing [36]. Y. Li et al. developed a compact Cascade-Cas3 Dvu I-C system with Cas11c, finding it to effectively produce stable transgenic lines in maize and rice, with an editing efficiency as high as 86.67% [78]. Studies have shown that using the Dvu type I-C system and crRNA paired within the PAM in HEK293T cells, the editing efficiencies for the *HPRT*, *AAVS1*, and *HEK3* genes were approximately 54.6%, 46.72%, and 2.89%, respectively. For the human *HPRT* gene, a DNA fragment replacement of about 600 base pairs was achieved with an efficiency of 18.46% through homology-directed repair (HDR). Furthermore, base editors were developed: Dvu-ABE (adenine base editor) induced A-to-G conversions at multiple endogenous gene sites with an efficiency of up to 61.63%, while Dvu-CBE (cytosine base editor) induced C-to-T conversions with an efficiency of up to 57.08% [79]. Due to the system’s ability to produce highly efficient large-fragment deletions, unwanted adverse editing sometimes occurs. Researchers discovered that AcrIC8 and AcrIC9 can act as potent inhibitors in mammalian gene editing; AcrIC8 inhibits PAM recognition through allosteric regulation, while AcrIC9 achieves inhibition by directly competing with the PAM binding site, thereby reducing off-target effects and enhancing the specificity of gene editing [80].

### 4.4. I-D CRISPR System

The type I-D CRISPR-Cas system is a hybrid system typically found in cyanobacteria and archaeal species [5,81,82,83], encoding proteins such as Cas5 (Csc1), Cas7 (Csc2), Cas6, the helicase protein Cas3′, and the large subunit Cas10d [24,41,51,82]. Researchers demonstrated that the I-D effector complex requires a PAM, the helicase Cas3′, and the HD nuclease domain (Cas3″) embedded in Cas10d for cleavage of dsDNA. The cleavage patterns of non-target sites (NTS) and target sites (TS) are similar to those of other type I systems, and the I-D system possesses a unique dsDNA cleavage activity characteristic of other type I CRISPR-Cas systems, positioning it as an evolutionary intermediate between type I and type III systems [18,41,54,84]. The *Synechocystis* I-D system utilizes a 5′-GTN-3′ PAM sequence in vivo and in vitro, where “N” represents any nucleotide, while the PAM sequence of green membrane bacteria is slightly different, being 5′-GTH-3′, with “H” representing A, C, or T [85].

In the study of gene editing with the type I-D CRISPR-Cas system, K. Osakabe et al. developed a CRISPR TiD system that generates bidirectional deletion mutations and small insertions or deletions (indels) in the tomato genome with a relatively low off-target rate, highlighting a unique feature and advantage of the I-D system compared to other systems [86]. Subsequently, researchers constructed eukaryotic expression vectors for type I-D Cas proteins, transfected them into mammalian cells, and targeted *EMX1* in HEK293T cells to produce double-strand breaks (DSBs) and small indels with mutation rates of 19.6% and 12.5% [87]. The I-D system produced a spectrum of bidirectional long chromosomal deletions ranging from 2.5 to 19 kb in both types of eukaryotic cells, with editing efficiencies of up to 57%, consistent with the in vitro cleavage patterns of the I-D system [41]. Moreover, recent studies on the I-D systems of *S. islandicus* and *Synechocystis* have shown that the hybrid characteristics of the I-D complex will allow binding to dsDNA, ssDNA, and single-stranded RNA (ssRNA) [41,88,89]. Further research on the type I-D CRISPR-Cas system is needed to develop more gene-editing tools and enrich the CRISPR-Cas toolbox.

### 4.5. I-E CRISPR System

The type I-E CRISPR-Cas system, derived from *Escherichia coli* K12, comprises the genes *Cas1*, *Cas2*, *Cas3*, *Cse1*, *Cse2*, *Cas7*, *Cas5*, and *Cas6e*, with the latter five encoding proteins that constitute the Cascade [90,91,92]. The structure can be broadly divided into a helical scaffold composed of Cas5e, Cas7 (1–6), the crRNA-processing enzyme Cas6e, and an abdominal region made up of Cse1 and two Cse2 proteins [93]. Unlike other type I CRISPR systems, the I-E system lacks Cas4 [94]. The highly conserved Cas proteins Cas1 and Cas2 form a complex (Cas1–2 complex) that captures protospacer elements and facilitates their integration into the CRISPR array. Cas1-Cas2 acts as a sequence-specific integrase [95,96,97]. In *E. coli*, the Cas6e protein plays a role in stabilizing the interaction between crRNA and Cascade, but this role is secondary; CRISPR interference and adaptation can occur without Cas6e [98].

In the study of gene editing in prokaryotes, Kiro, R et al. successfully knocked out two genes of the T7 phage by targeting specific genes in the T7 phage genome using the I-E-type CRISPR-Cas system of *E. coli*, a method that can be easily applied to any phage genome with only minor modifications [99]. Similarly, A. M et al. accomplished efficient editing of the Vibrio cholerae lysogenic phage genome using the I-E CRISPR-Cas system, which successfully produced small (33 bp) and large (>2.6 kb) gene deletions, as well as gene substitutions [100]. K. N. Yoganand et al. utilized the I-E CRISPR-Cas system in the polyploid radiation-resistant bacterium *Deinococcus radiodurans* to target phoN, achieving approximately 90% knockout efficiency and effective gene silencing [101]. Z. Qin et al., through analysis of the *G. oxydans* WSH-003 genome sequence, identified a typical I-E-type CRISPR/Cas system and developed it into a CRISPRi system by expressing the corresponding crRNAs, successfully achieving multiplex gene repression [102]. This system holds promise for future genetic modification of other genetically recalcitrant strains.

In the application of genome editing in eukaryotic cells, J. K et al. pioneered the use of the I-E-type CRISPR-Cas system in plant cells, successfully activating a chromosomal gene responsible for anthocyanin biosynthesis, thereby disrupting the color phenotype of corn embryo cells [103]. Cas3, endowed with helicase and single-stranded DNA nuclease activities, can degrade DNA continuously after cutting the target DNA. This characteristic of Cas3, as opposed to the single base mutation capability of Cas9, offers new avenues for the field of gene editing. The I-E-type CRISPR-Cas system from *Thermobifida fusca*, introduced via electroporation of Cascade and Cas3 proteins, has induced a series of large genomic deletions in human cells. Upstream of a single target site, the effector complex causes gene silencing by inducing long-segment DNA damage (ranging from several hundred bases to 100 kb), with an efficiency of up to 60%, thereby demonstrating their potential for large-scale genomic manipulations [104]. Researchers also developed effector complexes based on transfected plasmids and heterologous expression of the Cas3 protein in human cells, facilitating targeting at different locations with an editing range of up to 200 kb [26]. Hiroyuki Morisak et al. demonstrated that the I-E-type CRISPR mediates distinct DNA-cutting activities in human cells. Notably, Cas3, with its helicase and nuclease activities, primarily triggers deletions of thousands of base pairs upstream of the 5′-ARG PAM without significant off-target activity. This directed, and extensive DNA degradation mediated by Cas3 can be harnessed for the introduction of functional gene knockouts and knock-ins. As an example of potential therapeutic applications, researchers showcased Cas3-mediated cutting of the *DMD* gene exon in patient-induced pluripotent stem cells (iPSCs) [34]. Furthermore, it was shown that the endonuclease inactivation of Cas9 (dCas9) can precisely control large-fragment deletions mediated by Cas3 in mammalian cells. They found that dCas9-controlled CRISPR-Cas3-mediated precise 1 kb segment deletion efficiency was 94.52% in *DMD* and 75.89% in *ERCC4*, while the efficiency for precise 2 kb deletion was 98.49% and 73.91%, respectively, and finally achieved precise Y chromosome elimination in mice [105]. These findings broaden our understanding of the class 1 CRISPR system, suggesting their potential as a unique genome-editing tool in eukaryotic cells and a stable and controllable precision gene-editing tool.

### 4.6. I-F CRISPR System

Among all type I CRISPR-Cas systems, the type I-F CRISPR system is one of the most complex Cas protein compositions and most elaborate mechanism of action subtypes discovered to date, featuring two key ingredients: the crRNA-guided surveillance complex (Csy complex) and the Cas3 protein with helicase–endonuclease activity [106,107,108]. Research indicates that the Cas1 protein can interact with a protein containing a Cas2 domain (Cas2–3) to form a complex involved in the adaptation process of the I-F-type CRISPR-Cas system in *Acidovorax avenae* [106]. Subsequent studies on the Cas1 protein from the I-F-type CRISPR system of *P. atrosepticum* revealed characteristic folding through X-ray crystallography but also exposed significant structural plasticity of a conserved loop unique to I-F-type Cas1 proteins, resulting in a highly asymmetric Cas1 dimer. Furthermore, it was discovered that Cas1 is crucial for the adaptation of the I-F-type CRISPR-Cas system and relies on a conserved aspartic acid residue [109]. In *Pseudomonas aeruginosa*, the I-F-type CRISPR-Cas immune process assembles the Cas6f-crRNA complex with six Cas7f and one each of Cas5f and Cas8f to form the Csy complex, which recognizes invading DNA via PAM sequences and binds complementarily. Once the Csy complex binds to the invading DNA, the Csy-DNA complex recruits the Cas3 protein to cleave and ultimately degrade the DNA [110].

It was reported that the I-F-type CRISPR-Cas system can target genomic DNA. To test whether the I-F subtype RNA targeting activity could provide immunity against RNA phage infection, I-F CRISPRs were designed to target the RNA genome of the MS2 phage, and experiments ultimately proved that the I-F-type CRISPR-Cas system could detect and destroy dsDNA guided by crRNA but could not destroy RNA [111]. In one study, researchers utilized the I-F-type CRISPR-Cas system in *Zymomonas mobilis* ZM4 to establish an efficient I-F-type CRISPR-based genome-editing toolkit, achieving various genome engineering goals, including gene deletion and replacement (100% efficiency), in situ modification (100%), large-fragment (greater than 10 kb) deletion (50%), and simultaneous multi-gene editing (18.75%). Moreover, through the programming of the I-F system, efficient gene repression was also achieved [112]. In another study, researchers used the endogenous I-F-type CRISPR-Cas system to complete efficient genome editing of clinical and environmental isolates of the prototypic MDR pathogen *P. aeruginosa* [113]. What is more, S. Yao et al. developed a single-plasmid-mediated gene-editing tool for gene editing and gene regulation in *Acinetobacter baumannii* using the endogenous type I-F CRISPR-Cas system and significantly increased the knockout rate from 12.5% to 75.0% by introducing the RecAb homologous recombination system [114].

In addition, Y. Chen et al. demonstrated that the type I-F CRISPR-Cas system can be reprogrammed to activate endogenous gene expression in human cells, and the fusion of the Csy3 subunit of the type I-F PaeCascade with the transcriptional structural domain (VPR) resulted in the generation of crRNA-dependent reporter genes and the activation of endogenous genes, which broadens the use of CRISPR systems as gene regulation tool in mammalian cells [115]. This study expands the application of CRISPR systems as gene regulation tools in mammalian cells, highlighting their potential in therapeutic gene modulation. A research team led by Xiang Hua from the Chinese Academy of Sciences has discovered that the I-F2-type system from *Moraxella osloensis* (Mos350) exhibits significant activity in eukaryotic cells. By fusing the VPR transcriptional activator to the system’s Cas7 protein, the team achieved upregulation of multiple genes. Additionally, they enhanced transcriptional levels by extending the spacer to increase the copy number of Cas7-VPR fusion proteins within the Cascade complex. Finally, they developed a base-editing tool with a broad editing window, achieving over 50% editing efficiency, which also demonstrated potential at disease-relevant loci [116].

Research on anti-CRISPR proteins (Acrs) of the I-F-type system has been a hot topic in recent years. In the I-F-type system, AcrIF1, AcrIF2, AcrIF4, AcrIF6, AcrIF7, AcrIF8, AcrIF9, AcrIF10, AcrIF14, and AcrIF24 exert their inhibitory effects by directly binding to the Csy complex, preventing it from binding to DNA [117,118,119,120,121,122], thereby resisting the CRISPR system. However, AcrIF23 inhibits the nuclease activity of Cas2/3 by binding to it without impeding the recruitment of Cas2/3 to the Csy complex [123]. Currently, there are still a few cases of successfully applying the I-F-type CRISPR-Cas system to the genome editing of eukaryotic organisms, and further development and optimization of the system are needed to make it a powerful gene-editing tool in the field of genome editing in the future.

### 4.7. I-U CRISPR System

The type I-U CRISPR-Cas system was first identified in *Geobacter sulfurreducens* [13]. Researchers consider the I-U system to be a subtype of the I-B system, sharing the same protein composition with the I-B system, where the cas8 gene is a characteristic gene of the I-B system [51]. In the I-U system, it is named Cas8u, and this subtype is now also referred to as the I-G type. The main feature of this system is that its fused Cas4 and Cas1 genes can introduce functional spacers carrying the “TTN” PAM sequence at a higher frequency [13]. Additionally, L. Ou et al. analyzed the I-U system in Bifidobacterium and demonstrated that its fused Cas4 and Cas1 genes play an important role in the evolution of the strain [124]. Currently, there is a scarcity of research related to the gene-editing applications of the I-U (I-G)-type CRISPR-Cas system. In recent related research, Q. Shangguan et al. utilized the I-G-type CRISPR system from *Thioalkalivibrio sulfidiphilus* to edit the *Escherichia coli* genome, targeting the *lacZ* gene and observing long-range bidirectional deletions (10 to 55 kb) [125]. The development of gene-editing applications for the I-U CRISPR-Cas system requires further in-depth exploration. Researchers need to further elucidate its mechanism of action and repurpose it as a programmable, efficient gene-editing tool for eukaryotic organisms (Table 4).

### 4.8. Transposon-Associated Type I CRISPR Systems

Transposon-associated CRISPR-Cas systems are a relatively recent discovery and have been the subject of intensive research in recent years. Researchers, through exploration of bacterial and archaeal genomes, have identified numerous Tn7-like transposons that incorporate I-F-type and I-B-type CRISPR-Cas systems. These systems can guide the directional transposition of RNA, thereby facilitating the dissemination of genetic elements through bacteriophages and plasmids [127,128]. J. Strecker et al. discovered a CRISPR-associated transposase (CAST) from the cyanobacterium *Scytonema hofmanni*, which catalyzes RNA-guided DNA transposition by inserting DNA fragments 60 to 66 bp downstream of the protospacer adjacent to the single protospacer. They demonstrated that this transposase can be reprogrammed to integrate DNA into targeted sites in the *Escherichia coli* genome with a high frequency of up to 80% [129]. However, this system is composed of Tn7-like transposase subunits and a V-K-type CRISPR effector (Cas12k).

Furthermore, S. E. Klompe et al. reported the molecular mechanism of programmable transposition of the I-F-type CRISPR-Cas system from Vibrio cholerae Tn6677 in *E. coli*, establishing the INTEGRATE (Insertion of Transposon Elements by Guided RNA) system. Its targeting function is provided by a complex between the type I CRISPR effector complex Cascade and the transposase protein TniQ, demonstrating highly specific, whole-genome DNA insertions at multiple unique target sites [29]. Notably, as an I-F-type CRISPR system, the INTEGRATE system does not require tracrRNA, making it more suitable for direct insertion of the same DNA fragment across multiple genomic targets compared to the CAST system.

Additionally, in 2021, P. L. H. Vo et al. optimized the INTEGRATE system, achieving precise DNA insertion of up to 10 kb in bacteria with an efficiency of approximately 100%. They also utilized multi-spacer CRISPR arrays to simultaneously insert at three genomic sites and combined orthogonal integrases and recombinases to achieve multi-site knockouts [130]. This research reveals that the INTEGRATE system can serve as a powerful tool for multi-target, large-fragment genome editing. However, a recent study discovered that the CAST system can achieve targeted DNA integration in human cells, enabling precise, large-fragment insertions without relying on double-strand breaks. Notably, during this process, the researchers identified the effector protein ClpX, which facilitates the disassociation of the transposase, thereby enhancing integration efficiency through continuous optimization [131]. In addition, with the advancement of biological research technologies, further studies should delve deeper into the structural mechanisms of transposon-associated CRISPR-Cas systems and design more convenient and efficient genome-editing tools for utilization.

### 4.9. Experimental Workflow of Type I CRISPR-Cas Systems 

**Figure 4:**
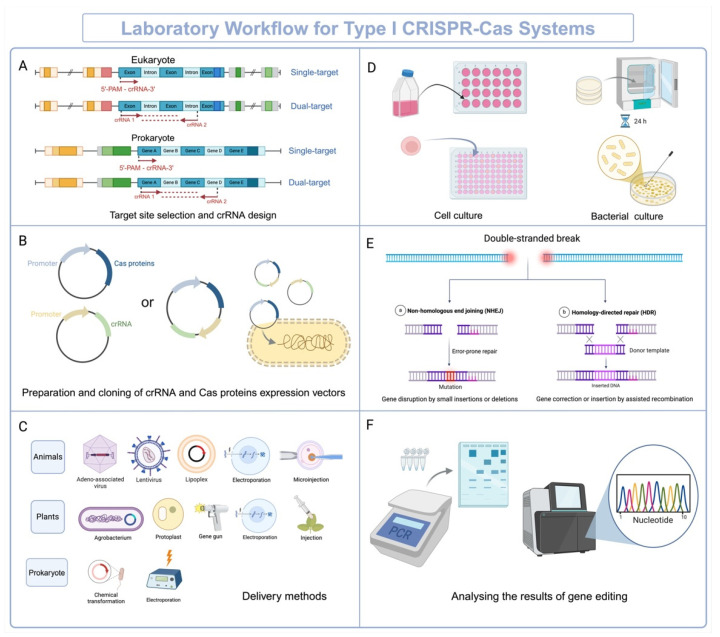
Laboratory workflow for type I CRISPR-Cas systems: This schematic illustrates the major steps in the laboratory workflow for utilizing type I CRISPR-Cas systems. (**A**) Target site selection and crRNA design: In eukaryotic and prokaryotic systems, single-target or dual-target approaches are employed, with crRNA designed to include the spacer sequence complementary to the target DNA and PAM sequences, such as I-E 5’-PAM (e.g., 5’-AAG-3’). Small rectangles preceding and following the target regions represent non-coding sequences: upstream regions may include promoters, enhancers, or regulatory elements essential for transcription initiation, while downstream regions may consist of transcription terminators or untranslated regions (UTRs) influencing mRNA stability and translation. (**B**) Preparation and cloning of crRNA and Cas protein expression vectors: crRNA and Cas protein expression are driven by appropriate promoters, encoded either on a single-plasmid or dual-plasmid system for modular flexibility. (**C**) Delivery methods: Delivery strategies vary based on the organism. For animals, options include viral vectors, lipofection, or electroporation; for plants, methods include Agrobacterium-mediated transformation, protoplast transfection, and biolistic gene gun delivery; for prokaryotes, chemical transformation or electroporation is commonly used. (**D**) Cells or bacterial colonies are cultured and prepared for downstream processes. To ensure uniformity of the edited populations, monoclonal cells or single bacterial colonies are isolated and screened. (**E**) Editing mechanisms: The CRISPR-Cas system induces double-stranded DNA breaks, which are repaired by non-homologous end joining (NHEJ) or homology-directed repair (HDR), leading to desired edits. (**F**) Analysis of gene-editing results: Gene-editing outcomes are verified using PCR, gel electrophoresis, sequencing, or phenotypic analyses, ensuring the desired modifications are achieved.

The in vitro manipulation and validation of type I CRISPR-Cas systems involve several critical steps. These systems are characterized by their complex multi-protein Cascade complexes and the signature Cas3 protein, necessitating meticulous optimization and validation to ensure successful application in genome editing, interference, or other purposes. Researchers can select the appropriate system based on their experimental objectives and the specific features of each subtype, followed by a detailed experimental design. The following summarizes its workflow and validation methods (Figure 4).

The first step is designing the crRNA, which is crucial for guiding the Cascade complex to the target DNA. The design should align with the experimental objectives and the biological system under investigation. In eukaryotic systems, crRNAs are typically designed to target exonic or intronic regions, depending on whether the goal is functional gene knockout, splicing modification, or regulatory studies. Priority can be given to specific exons based on their biological significance. In prokaryotic systems, gene targets should be selected based on the genomic region of interest, ensuring compatibility with the Cascade complex and PAM sequence requirements, which are critical for all CRISPR-Cas systems. For instance, the I-E system recognizes the 5’-AAG-3’ PAM, and this sequence must be included in the design to ensure efficient targeting. Moreover, in type I CRISPR-Cas systems, crRNAs with a length of 32–34 bp have demonstrated higher efficiency. Additionally, depending on the experimental objective, single-target or dual-target strategies can be employed. Single-target approaches are generally used for simple gene-editing tasks, whereas dual-target designs allow for simultaneous targeting of multiple loci, enabling large-fragment deletions or the precise manipulation of complex genomic regions.

The next step is the construction of expression vectors. In eukaryotic systems, commonly used promoters such as CMV and EF-1α are employed to achieve high-level expression. In plant systems, plant-specific promoters like CaMV35S are typically utilized. The expression of crRNA also requires appropriate promoters; in eukaryotic systems, the U6 promoter is frequently used for efficient transcription, while in prokaryotic systems, promoters such as T7 or lac are selected to align with the target species’ transcriptional machinery. Expression vector design can adopt either a single-plasmid or dual-plasmid system. The single-plasmid system encodes crRNA, the Cascade complex, and the Cas3 protein on the same vector, simplifying the delivery process but imposing higher demands on vector capacity. In contrast, the dual-plasmid system separates the expression of crRNA and Cas proteins: one plasmid drives crRNA expression, while the other expresses Cascade and Cas3. This modular design allows for flexible optimization of the expression levels of each component, enhancing genome-editing efficiency while reducing off-target effects.

Third, delivery methods are crucial for ensuring the successful introduction of CRISPR-Cas components into cells or organisms. In eukaryotic cells, common methods include liposome-mediated transfection, electroporation, and viral vector-mediated transduction. The choice of method depends on the cell type and experimental requirements. For instance, lentiviral vectors are suitable for long-term studies requiring stable integration, while non-viral methods such as liposome-mediated transfection are more appropriate for transient expression. In plant systems, in addition to the commonly used Agrobacterium-mediated transformation and gene gun methods, protoplast transformation is also an efficient delivery strategy, especially for gene function studies and transient expression experiments. In animal models, targeted gene delivery can be achieved through microinjection or adeno-associated virus (AAV) vectors. In prokaryotic systems, electroporation or chemical transformation are the primary methods for plasmid delivery into cells, facilitating efficient genome editing or functional studies. After delivery, selection for homogeneity is a key step to ensure editing efficiency and specificity. For eukaryotic cells, fluorescence marker selection, antibiotic resistance selection, or flow cytometry sorting of monoclonal cell populations are commonly employed. In plants, selection can be performed using resistance markers or PCR to identify transformed plants or edited protoplasts. In prokaryotic systems, colony PCR is typically used to confirm successful editing. Additionally, for animal studies, genome editing can be performed via systemic or localized delivery methods (e.g., tail vein injection or targeted tissue injection), with genotype analysis and phenotype assessment verifying the editing outcomes. 

Then, the CRISPR-Cas system performs targeted DNA cleavage, which is subsequently repaired by the cell’s DNA repair mechanisms. Guided by Cas3, the Cascade complex binds to crRNA and targets specific DNA sequences for the degradation of large DNA fragments. DNA repair mechanisms, such as non-homologous end joining (NHEJ) or the less commonly used homologous directed repair (HDR), repair the DNA damage. NHEJ typically results in small insertions or deletions (indels), while HDR allows for precise sequence modifications when a repair template is provided.

Validation of editing outcomes is a critical step for assessing the efficiency and precision of type I CRISPR-Cas systems. Initial validation involves PCR amplification of the target region to detect deletions, followed by gel electrophoresis and Sanger sequencing for confirmation. For more precise quantification, digital PCR (ddPCR) can measure editing efficiency and deletion sizes with high accuracy. Next-generation sequencing (NGS) offers comprehensive insights into DNA degradation patterns and allows the detection of off-target effects. Long-read sequencing technologies, such as nanopore sequencing, are particularly valuable for characterizing the size and boundaries of large-fragment deletions induced by Cas3.

## 5. Conclusions and Prospect

The type I CRISPR-Cas system is the largest and most extensively studied of the class 1 systems, and its effector complexes have a wide range of applications in all aspects of gene editing. By modifying and optimizing the effector complexes of the type I CRISPR-Cas system, researchers can achieve precise knockouts, insertions, or replacements of target gene sequences. The CRISPR-Cas system will be refined and expanded upon in the future to boost the efficiency of gene editing and the functionality of its own effector complexes. 

Through extensive research on the type I CRISPR-Cas system, it has been discovered that its distinction from class 2 systems lies not only in the composition of its effector complex but also in its unique ability to induce large-fragment deletions. This ability to generate extensive genomic deletions could facilitate the study of non-coding regions and holds promise for the development of tools for large-scale functional genomic analysis. Although the complexity of its effector module may mean that the type I system might never match the practicality of class 2 systems in molecular applications, it appears to have potential in specific use cases. For example, by optimizing reaction temperatures to enhance Cas protein activity, it may become more suitable for application in human cells. Notably, a single crRNA has been shown to induce genomic deletions as large as 424 kb, with the ability to generate bidirectional deletions. However, when selecting target sites, it is important to consider the potential randomness of large-fragment deletions and their impact on essential genes required for cell survival, which could compromise cellular viability.

To achieve high efficiency in type I CRISPR-Cas systems, several key factors need to be considered:

1. PAM Sequence Selection: The efficiency of the type I CRISPR-Cas system heavily depends on specific PAM sequences. For instance, subtypes I-B demonstrate activity across various PAM sequences such as TTC, ACT, TAA, TAT, TAG, and CAC. This PAM dependency creates challenges in selecting target sites but also offers opportunities for optimization. By studying and screening different PAM sequences, researchers can significantly enhance the system’s targeting efficiency;
2. crRNA Optimization: The effector complexes of the type I CRISPR system are more complex than those of Cas9, requiring more precise crRNA design to ensure targeting accuracy and high system efficiency. Mismatched crRNAs can significantly reduce targeting efficiency and even lead to off-target effects [132]. By improving crRNA design and structure, researchers can ensure that the system identifies and binds to the target DNA with greater efficiency;
3. Temperature and Reaction Condition Optimization: The activity of type I systems, particularly Cas3 nuclease, is temperature-dependent. Different bacterial systems may require distinct reaction conditions to achieve optimal editing efficiency. Therefore, optimizing temperature and reaction conditions is crucial for improving the gene-editing efficiency of type I systems in non-native environments;
4. Cas Protein Expression: In heterologous systems, such as eukaryotic cells or non-native bacterial systems, the efficient expression of multiple Cas proteins is necessary to achieve effective gene editing. Enhancing Cas protein expression levels and ensuring their stability in host cells can further improve the performance of the type I CRISPR-Cas system.

However, I-type CRISPR-Cas systems also have several limitations:

1. Complexity of Effector Complexes: The effector modules of I-type CRISPR-Cas systems are more complex than those of class II systems (e.g., Cas9), involving multiple proteins. This complexity presents additional challenges in the simplification and application of these molecular tools. Such intricacy may limit their practicality in certain applications, particularly in high-throughput or cost-sensitive experimental settings;
2. Off-Target Effects: Despite the high specificity of I-type systems, the complexity of their multi-protein effector complexes can lead to off-target effects if targeting is not precise, resulting in unintended genomic modifications. Off-target effects pose a significant threat to the precision and safety of genome editing, especially in biomedical applications;
3. Target Site Restrictions: The selection of target sites in I-type systems is strictly constrained by PAM sequences, which limits their application range across different genomic environments. This restriction in PAM sequence recognition makes I-type systems less flexible in complex genomes compared to class II systems;
4. Challenges in Eukaryotic Applications: Achieving efficient genome editing in eukaryotic cells presents significant challenges for type I systems due to the complexity of delivering multiple components. Precise delivery methods are required to ensure that each effector protein reaches the target cells effectively. Although recent advancements in CRISPR system delivery—such as viral vectors, lipid nanoparticles, and polymeric carriers—show promise, further optimization is necessary for type I systems to improve delivery efficiency and minimize cytotoxicity.

In addition, precise control over the intracellular ratios of various effector components is crucial for maintaining editing specificity and reducing off-target effects. For instance, maintaining an optimal ratio between Cas proteins and crRNA can enhance targeting accuracy and reduce unintended genomic modifications. Future strategies may include the use of advanced delivery systems or tunable promoters to precisely regulate component expression in eukaryotic cells, thereby enhancing editing efficacy and safety.

Given these limitations, future research needs to address several scientific challenges: First, further investigation into the mechanisms of off-target effects in I-type systems is essential to develop more precise targeting tools and reduce unintended genomic editing events. Understanding the molecular interactions between crRNA and target sequences is crucial for enhancing editing specificity and safety. Second, simplifying and optimizing the effector complexes of I-type systems will improve their practicality across various applications. This includes reducing the complexity of multi-protein complexes or enhancing their efficiency through protein engineering. Finally, expanding the PAM sequence recognition capabilities of I-type systems will significantly increase their targeting range within genomes, thereby improving their application flexibility. High-throughput screening and computational modeling to discover and design new PAM sequences will be key areas of focus for future research. 

As CRISPR technology, including the type I CRISPR-Cas system, continues to develop and find applications in fields such as gene therapy and environmental engineering, ethical and biosafety concerns are becoming increasingly important. In gene therapy, editing the human genome raises questions about long-term effects, off-target impacts, and potential unintended consequences, which require strict regulatory frameworks, as well as careful considerations for patient consent and safety. In environmental engineering applications, such as genetically modifying organisms to achieve ecological benefits, there are risks of unintended environmental impacts or gene transfer to wild populations, posing challenges for control and monitoring. Therefore, while the type I CRISPR system offers a promising tool for genome editing, future research should also address these ethical and biosafety issues to ensure responsible scientific and technological applications. Open debates and transparency are essential to foster broad discussions and regulatory oversight for CRISPR technology.

**Figure 5:**
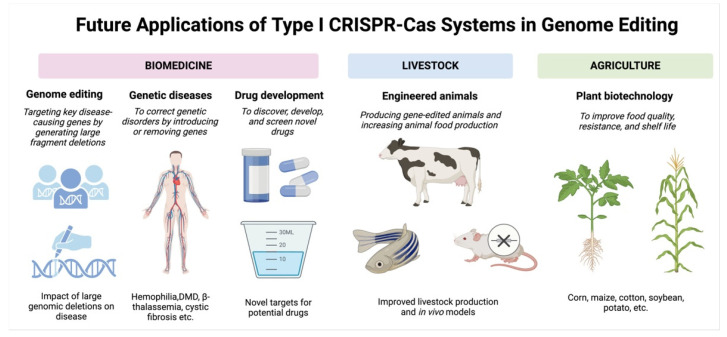
Future application areas for the type I CRISPR-Cas system: This graphic illustrates potential future applications of the type I CRISPR-Cas system. Three key areas could benefit from this technology: first, in biomedicine, where it could enable genome editing to target disease-causing genes, facilitate the treatment of genetic disorders (such as hemophilia, Duchenne muscular dystrophy, and cystic fibrosis), and aid in drug development by identifying novel therapeutic targets; second, in animal husbandry, where it could be employed to breed genetically modified animals and enhance animal food production; and third, in agriculture, where it could be used to improve the quality, resistance, and shelf life of agricultural products.

The effector complex of the type I CRISPR-Cas system shows great potential for various aspects of genome editing and could be a valuable tool in future genetic research (Table 5). Its features and functions can offer more methods and ideas in the fields of nucleic acid detection, genome function research, genome modification, creation of animal or cell models, as well as disease treatment, plant and animal breeding, and drug delivery based on the principles of CRISPR gene editing (Figure 5).

## Figures and Tables

**Table 1:** Protein composition of the type I CRISPR system’s isoforms.

Subtype	Composition
I-A	Cas1, Cas2, Cas4, Cas5, Cas6, Cas7, Cas8a, Cas11, Cas3′, Cas3″
I-B	Cas1, Cas4, Cas5, Cas6, Cas7, Cas8b1, Cas3
I-C	Cas1, Cas4, Cas5, Cas7, Cas8c, Cas11, Cas3
I-D	Cas1, Cas4, Cas5, Cas6, Cas7, Cas3, Cas3’’, Cas10d
I-E	Cas1, Cas2, Cas5, Cas6, Cas7, Cas8e, Cas11, Cas3
I-F	Cas1, Cas2, Cas5, Cas6, Cas7, Cas8f, Cas3
I-U	Cas1, Cas2, Cas4, Cas5, Cas6, Cas7, Cas8u, Cas3

**Table 2:** Function of Cas proteins in the type I CRISPR-Cas system.

Protein	Function	References
Cas1/Cas2	Recognize and integrate the protospacer sequences	[9]
Cas4	Ensure that DNA sequences flanking only the PAM are inserted into the host DNA	[11]
Cas5	Fix the 5′end of crRNA in the Cascade, and in the type I-C system, mature the pre-crRNA	[23,25]
Cas6	Recognize and cleave the hairpin motif within the pre-crRNA, fix the crRNA’s 3′ end in the Cascade	[24]
Cas7	Binds crRNA and forms the backbone structure of the Cascade complex	[34]
Cas8	Identifying PAM sequences and stabilizing the spatial position of Cas3	[35]
Cas11	Forming the belly of the Cascade and stabilizing crRNA and R-loop structures	[36]
Cas3	Acts as a nuclease and helicase and is responsible for cutting DNA	[8,22]

**Table 3:** Comparison of key CRISPR-Cas systems: type I, Cas9, Cas12, and Cas13.

System Type	Features	Advantages	Limitations	References
Type I (I-A, I-B, I-C, I-D, I-E, I-F, I-U)	Targets DNA, uses a multi-protein complex called Cascade, requires PAM sequence	The multi-protein effector complexes allow for more flexible regulation in both prokaryotic and eukaryotic organisms, large-scale DNA deletions, bidirectional deletions	Complexity of effector complexes, off-target effects, target site restrictions	[27,36,39,40,41]
Type II (Cas9)	Primarily targets DNA, requires PAM sequence recognition	Simplified single-protein system, suitable for genome editing, transcriptional regulation, base editing	Prone to off-target effects, larger protein size, limited to small-scale editing	[42,43,44]
Type V (Cas12)	Primarily targets DNA, capable of single-strand DNA cleavage, collateral cleavage activity targeting nearby ssDNA	High sensitivity for nucleic acid detection, compact size for portable applications (Cas12f), flexible targeting without PAM requirement (Cas12f)	Risk of non-specific ssDNA degradation, crRNA-dependent targeting complexity	[45,46]
Type VI (Cas13)	Primarily targets RNA, specifically cleaves single-stranded RNA	No DNA damage, ideal for RNA editing and detection, specific RNA recognition	Not suitable for DNA editing, off-target effects, and RNA target specificity need improvement	[47,48]

**Table 4:** Gene-editing applications and efficiency of the type I CRISPR-Cas system.

Subtype	PAM (5’-)	Gene-Editing Applications and Efficiency	References
I-A	YCN	Nucleic acid detection, gene deletion efficiency up to 90%	[39]
I-B	TTC, ACT, TAA, TAT, TAG, CAC	Bacterial genome-editing efficiency reaches 100%, eukaryotic cell genome-editing efficiency reaches 41.2%	[62,69,70]
I-C	TTC	Bacterial genome-editing efficiency reaches 100%, gene-editing efficiency in the human genome reaches 95%, plant gene-editing efficiency reaches 86.67%	[27,36,75,78]
I-D	GTN, GTH	Bidirectional large-fragment genomic deletions with efficiencies up to 57%	[41,85]
I-E	AWG	Bacterial genome large-fragment deletion efficiency of up to 90%, human cell gene knockout efficiency reaches 60%, mammalian cell gene knockout efficiency as high as 98.49%	[101,104,105,126]
I-F	CC	Bacterial genome large-fragment deletion efficiency of up to 75% as a gene regulation tool in mammalian cells	[114,115,126]
I-U	TTN	Bidirectional large-scale genomic deletions in bacteria	[13,125]

N = A, C, G, T; W = A, T; Y = C, T; H = A, C, T.

**Table 5:** Biomedical applications of the type I CRISPR-Cas system.

Biomedical Applications of the Type I CRISPR-Cas System		References
Genome Editing	Achieves highly efficient genome editing in *Clostridium pasteurianum* using the I-B system, with success rates up to 100%.	[63]
	Employs a compact Cascade-Cas3 Dvu I-C system for producing stable transgenic lines in maize and rice, with editing efficiencies reaching 86.67%.	[78]
	Employs the I-E system in human cells to induce extensive genomic deletions for functional gene studies.	[104]
Gene Regulation	Suppresses transcription of target genes in bacteria using CRISPRi.	[102]
	Activates key genes in plants and microbes to enhance traits such as resistance and biosynthesis capabilities.	[103]
Potential Therapeutic Applications	Precisely edits pathogenic genomes to reduce virulence factor expression, advancing infectious disease research and treatment.	[76]
	Achieves precise exon cleavage with multiple exon skipping in DMD patient-derived iPSCs using the I-E system, restoring anti-myogenic protein expression.	[34,133]
	Employs CRISPRi to silence specific genes in the radiation-resistant bacterium *Deinococcus radiodurans*, facilitating research on radiation response.	[101]

## Data Availability

No new data were created or analyzed in this study.
